# The Role of Semaphorin 6D (Sema6D) in Non-Muscle-Invasive Bladder Cancer—A Preliminary Study on Human Plasma and Urine

**DOI:** 10.3390/biomedicines12071426

**Published:** 2024-06-27

**Authors:** Piotr Purpurowicz, Tomasz W. Kaminski, Władysław Kordan, Anna J. Korzekwa, Zbigniew Purpurowicz, Zbigniew Jabłonowski

**Affiliations:** 1 Department of Urology and Urological Oncology, Municipal Hospital in Olsztyn, 10-045 Olsztyn, Poland; purp8@wp.pl; 2 Pittsburgh Heart, Lung and Blood Vascular Medicine Institute, University of Pittsburgh, Pittsburgh, PA 15260, USA; tkaminski@versiti.org; 3 Thrombosis and Hemostasis Program, VERSITI Blood Research Institute, Milwaukee, WI 53226, USA; 4 Department of Animal Biochemistry and Biotechnology, Faculty of Animal Bioengineering, University of Warmia and Mazury in Olsztyn, 10-719 Olsztyn, Poland; wladyslaw.kordan@uwm.edu.pl; 5 Research Group of Biodiversity Protection, Institute of Animal Reproduction and Food Research of Polish Academy of Sciences in Olsztyn, 10-748 Olsztyn, Poland; a.korzekwa@pan.olsztyn.pl; 6 1st Department of Urology, Medical University of Lodz, 90-549 Lodz, Poland; zbigniew.jablonowski@umed.lodz.pl

**Keywords:** semaphorin 6D, sema6D, bladder cancer, urology

## Abstract

The incidence of bladder cancer worldwide in the last three decades has been increasing in both men and women. So far, there is no established non-invasive bladder cancer biomarker in daily clinical practice. Semaphorin 6D (sema6D) is a transmembrane protein that belongs to the class VI semaphorins. The aim of this study was to evaluate for the first time the potential role of sema6D in bladder cancer. The study group consisted of 40 patients with non-muscle-invasive bladder cancer (NMIBC) and the control group of 20 patients without malignancies. There was a statistically significantly higher urinary sema6D concentration in patients than controls (*p* < 0.05) but no significant difference in plasma 6D. There were no statistically significant differences in urinary or plasma concentration of sema6D between low- or high-grade cancer and according to the tumor stage in TNM classification. There was a statistically significant negative correlation between plasma sema6D and age of patients (R = −0.6; *p* = 0.019). Plasma sema6D does not seem to be useful in the clinical practice at this point. However, the urinary sema6D concentration could potentially serve as a marker of NMIBC used for diagnostic purposes, monitoring, and early relapse detection or the assessment of the treatment efficacy. Urinary sema6D is probably not associated with the grading or staging of NMIBC, so it cannot be used for the prediction of disease prognosis.

## 1. Introduction

The incidence of bladder cancer worldwide in the last three decades has been increasing in both men and women [1]. Bladder cancer ranks ninth in incidence worldwide [1]. In 2018, nearly 550,000 people worldwide were diagnosed with bladder cancer, with a higher incidence among males, who account for 77% of patients, while nearly 200,000 people died [2], whereas in 2020, approximately 212,536 people worldwide died [3]. Bladder cancer is the eighth most common cause of death related to cancer among men in the USA [3]. The epidemiological data presented here prove that bladder cancer is a significant problem in the world population. Its high incidence and, in addition, its biological features, namely the tendency to multiple recurrences and progression despite local therapy, lead to a significant burden on health care [4].

**Figure 1:**
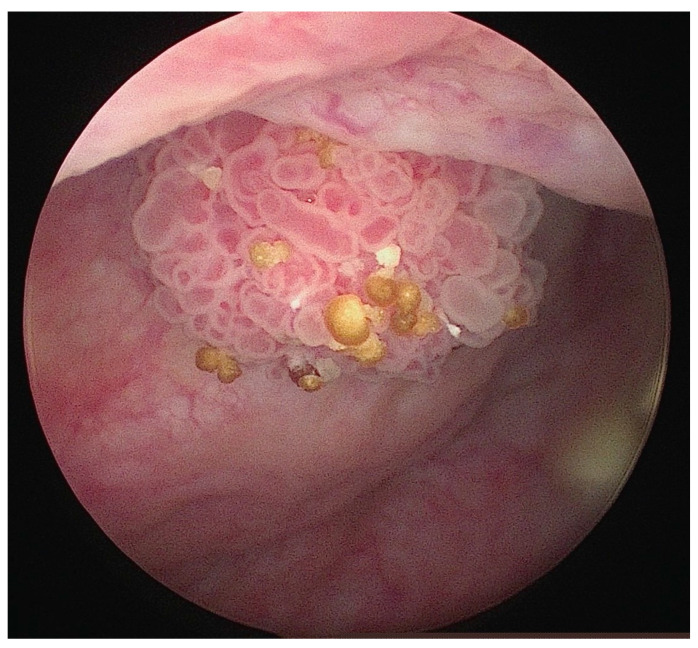
Bladder tumor visualized during cystoscopy.

Diagnosis of bladder cancer is based on the patient’s history, physical examination, as well as additional examinations (with particular emphasis on cystoscopy) (Figure 1) [5]. About 75% of patients with urothelial carcinoma of the bladder are diagnosed at the stage of non-muscle-invasive bladder cancer (NMIBC) [6,7]. The outcome of NMIBC is mainly expressed by the rates of local recurrence (15% to 61% at 1 year) [8] and progression (from 0.2% to 17% at 1 year) [9]. So far, there is no established bladder cancer biomarker that would be used in daily clinical practice.

Semaphorins are extracellular proteins with relay functions that are involved in many processes in the human body. There are eight classes of semaphorins. Each semaphorin has a cysteine-rich sema domain in its structure, which defines the properties of individual proteins. Semaphorins are involved in carcinogenesis, where they can serve both a promoting and inhibitory function. So far, they play a role in the pathogenesis of such cancers as lung cancer, ovarian cancer, breast cancer, endometrial cancer, oral squamous cell carcinoma, melanoma, prostate cancer, kidney cancer, and stomach cancer [10,11,12,13].

Yet, with regard to bladder cancer, only the role of semaphorin 3A and 4D has been studied. They have been shown to predispose to the development of this cancer. To date, the role of semaphorin 6D in bladder cancer has never been studied [14,15].

Class 6 of semaphorins comprises four subgroups: semaphorins A, B, C, and D [16]. Noteworthy, compared to other semaphorin types, the cytoplasmic parts of semaphorin 6 family members are longer. Semaphorins take part in the signal transmission due to interactions characterized by a low affinity, which start with the activation of specific plexin receptors by the sema domain. During the activation, the sema domain forms a structure where two plexin units bind, which leads to a formation of complex seven-bladed β-propeller-fold configuration. This compound acts as the connection point for mixed semaphorin–plexin complexes associated with the sema domains, leading to the formation of structures containing four different components. Among the different types of plexins, semaphorin class 6 initiates signal transmission by interacting with class A plexins (plexins A1–A4) [16].

Semaphorin 6D (sema6D) is a transmembrane protein that belongs to the class 6 semaphorins. It is associated with plexin A1 and acts as both a ligand and a receptor [17]. It has been reported to take part in the development of the heart [16,18] and retina [19]. As for non-cancerous diseases, sema6D is probably associated with liver fibrosis [16]. However, as for involvement in cancers, sema6D seems to exert the opposite impact regarding different types of cancers. It has been reported to act in a beneficial way in lung adenocarcinoma or breast cancer (triple-negative) [20]. On the other hand, it has a pro-tumor activity in case of gastric and colorectal cancer [20]. So far, in urology, sema6D has been explored in renal cell carcinoma, and the scientists concluded that it could become a diagnostic and prognostic biomarker for this cancer [20].

The aim of this study was to evaluate the potential role of semaphorin 6D in bladder cancer. To the best of our knowledge, it is the first experiment on sema6D in bladder cancer. It could potentially provide valuable information on its involvement in the development of this cancer, and the analyzed protein could perhaps become a potential marker of bladder cancer, which in the future could serve the purpose of early prevention in oncology and urology.

## 2. Materials and Methods

The study was performed in accordance with the principles of the Declaration of Helsinki [21] and was approved by the Bioethics Committee of the Medical University of Lodz, Poland (no. RNN/31/23/KE).

### 2.1. Study Population

The study group consisted of 40 patients (32 men, 8 women) with a mean age of 65.6 ± 1.46 years. The inclusion criteria were: age over 18 years, non-muscle-invasive bladder cancer (NMIBC) diagnosed for the first time, confirmed by the histopathological examination. The exclusion criteria were: age under 18 years old, pregnancy or breast-feeding, past history of malignant neoplasm (including NMIBC), muscle-invasive bladder cancer (MIBC), and immunosuppressants intake.

All patients, after the general anesthesia or subarachnoid spinal block, underwent transurethral resection of bladder tumor (TURBT) during which tumor tissue was obtained for histopathological examination to confirm bladder cancer and exclude patients with MIBC.

The control group consisted of 20 patients (17 men, 3 women) with a mean age of 64 ± 2.11 years. It was matched with the study group according to age, sex, and geographical region. The inclusion criteria applied were: age over 18 years, subjects without NMIBC or MIBC confirmed in cystoscopy, no history of any other malignancies, and no immunosuppressants intake.

The absence of bladder tumor in the control group was confirmed in cystoscopy during non-oncological procedures—transurethral resection of the prostate (TURP), ureterorenoscopic lithotripsy, or in negative cystoscopy in patients with suspicion of bladder tumor.

### 2.2. Urine and Blood Analysis

We collected samples of blood and urine at the beginning of the study both from the study and control groups. Fasting blood samples were collected using vacuum tubes. They were centrifugated for 10 min at 2000× *g*. Urine samples were collected as first morning specimens from mid-stream and centrifugated for 10 min at 2000× *g*. The obtained plasma and urine were stored at −80 °C until the analysis. Sema6D concentrations were measured by an enzyme-linked immunosorbent assay (ELISA) with commercially available kits according to the manufacturer’s protocol. Each run was performed in duplicate, and the average was considered as a single sample. Sema6D concentration was measured by the sema6D kit provided by Innovative Research Inc. (IHUSEMA6KT, Novi, MI, USA). The detection range was 31.2 pg/mL–2000 pg/mL, sensitivity 20 pg/mL, and absorbance was read at 450 nm length. All laboratory tests were performed by the same person in standardized laboratory settings.

### 2.3. Statistical Analysis

The distribution of the data was tested using the Shapiro–Wilk normality test. Normally distributed continuous data sets were shown as mean ± standard error of the mean (SEM). Comparisons between the two groups were conducted by *t*-test. Categorical variables were analyzed by the chi-squared test. The relationship between the paired variables was investigated by Spearman’s rank correlation. Multiple linear regression analysis was performed using a stepwise model with a forward elimination procedure to determine the combined influence of variables on particular parameters of the measured data set. A two-tailed *p*-value lower than 0.05 was considered statistically significant. Computations were performed using GraphPad 8 Prism (GraphPad Software; La Jolla, CA, USA).

## 3. Results

The study involved 40 patients with NMIBC and 20 subjects without malignant neoplasms as controls in total. Basic information about the participants is presented in Table 1.

There was no statistically significant difference between patients and controls in terms of sex, age, and GFR (*p* > 0.05).

Smokers were more prevalent among the patients’ group (*p* < 0.0001), and they used to smoke significantly more than the controls (*p* < 0.001).

**Figure 2:**
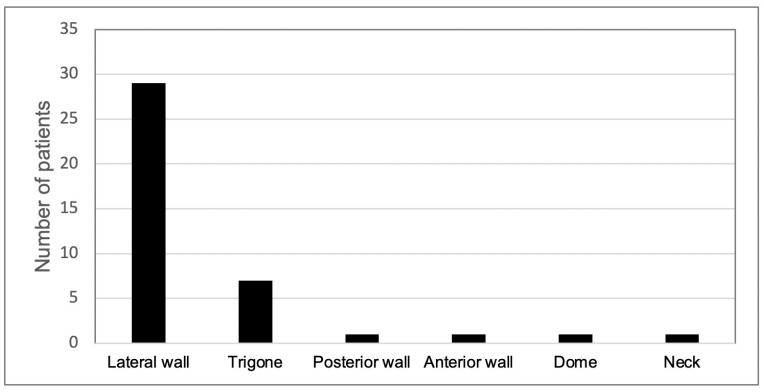
Localization of the tumors in the bladder.

The majority of patients (82.5%) had only one bladder tumor, whereas 17.5% had more than one lesion (2–4 lesions). Summarily, 70% of tumors were located on the lateral wall of the bladder, 17.5% in the trigone, 5% on the anterior wall, 2.5% on the posterior, 2.5% in the dome of the bladder, and 2.5% in the neck of the bladder (Figure 2).

All patients had a pure urothelial subtype of carcinoma diagnosed in pathology.

**Figure 3:**
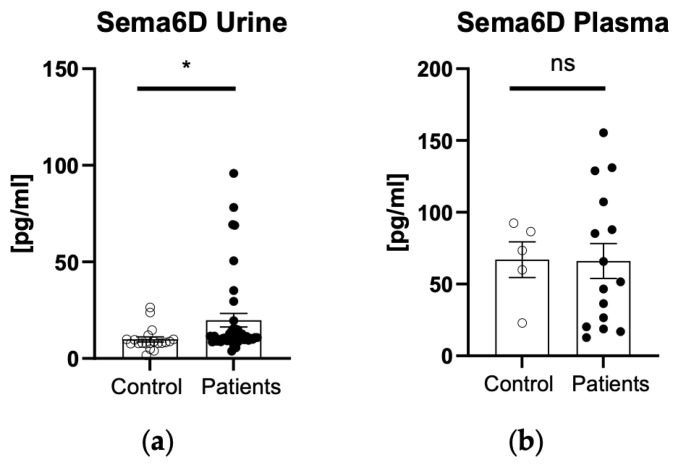
Urinary (**a**) and plasma (**b**) sema6D concentrations in patients and controls. * means statistically significant difference with *p* < 0.05; ns, non-significant.

There was a statistically significantly higher urinary sema6D concentration in patients than in controls (*p* < 0.05) (Figure 3a). There was no statistically significant difference in plasma 6D concentration between patients and controls (*p* > 0.05) (Figure 3b).

**Figure 4:**
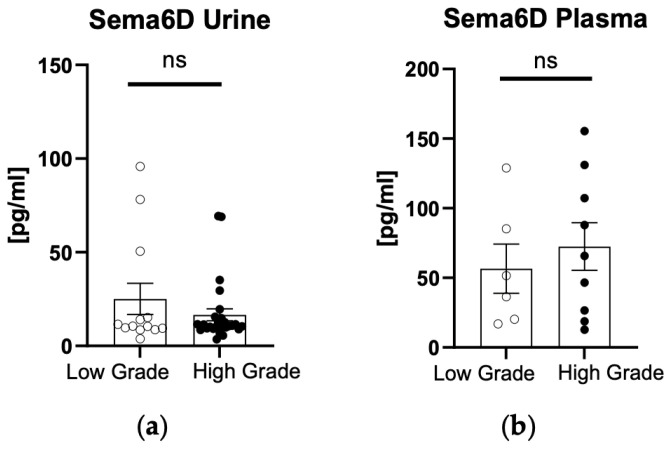
Urinary (**a**) and plasma (**b**) sema6D concentrations in patients and controls depending on the cancer grade. ns, non-significant.

After the division of patients into two groups—low- or high-grade cancer—there were no statistically significant differences in urinary nor plasma concentration of sema6D (*p* > 0.05) (Figure 4a,b).

**Figure 5:**
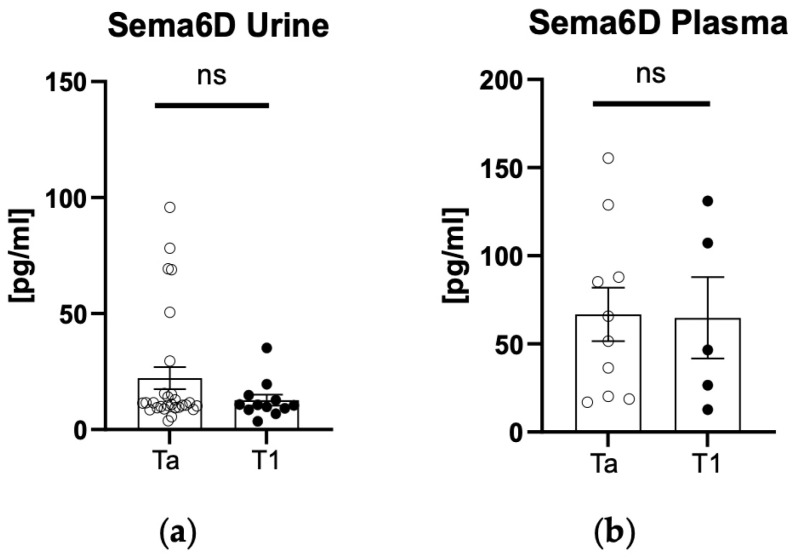
Urinary (**a**) and plasma (**b**) sema6D concentrations in patients and controls depending on the tumor stage. ns, non-significant.

Patients were also divided depending on the tumor stage in TNM classification. There were no statistically significant differences in urinary nor plasma concentration of sema6D with regard to tumor stage (*p* > 0.05) (Figure 5a,b).

**Figure 6:**
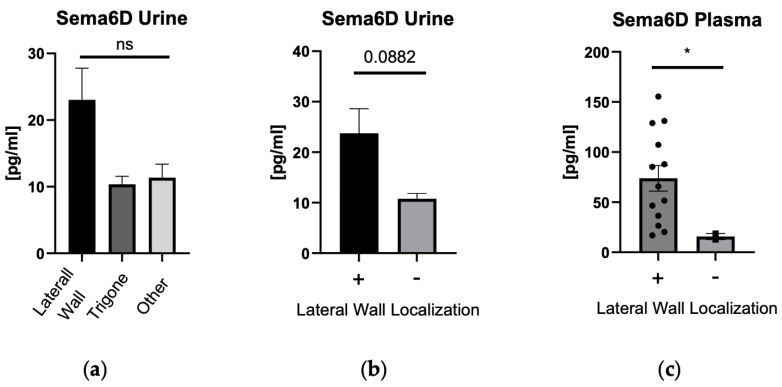
Sema6D concentration depending on the localization of the tumor in the bladder: in urine (**a**,**b**), in plasma (**c**). * means statistically significant difference with *p* < 0.05; ns, non-significant; (+) lateral wall; (−) other localization than lateral wall.

After the division of patients according to the localization of the tumor in the bladder, when we compared all points of origin with each other—there were no differences between sema6D concentrations (Figure 6a). However, when we compared the lateral wall (which was the most frequent site) to all the other localizations, we observed significantly higher sema6D concentrations in plasma of subjects with the lateral wall tumor (*p* < 0.05) and a strong upward trend (*p* = 0.088) for urinary sema6D concentration in subjects with the lateral wall tumor (Figure 6b,c).

**Figure 7:**
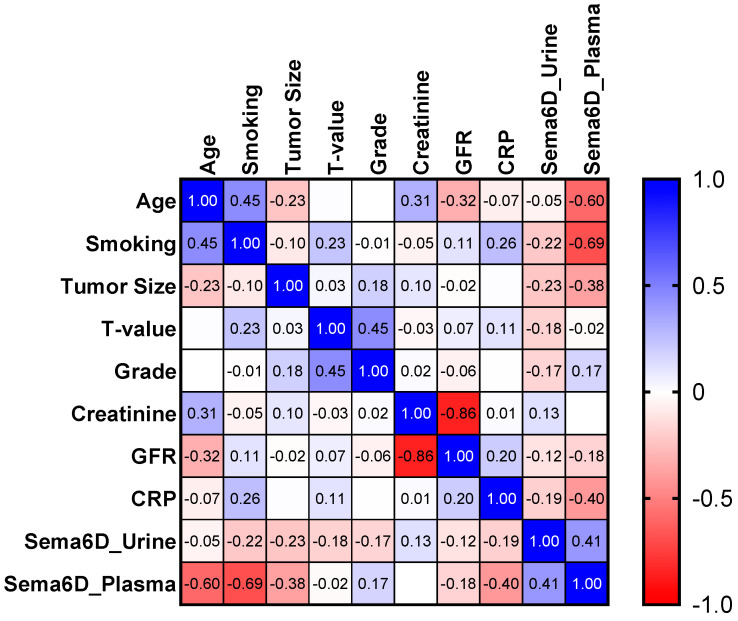
Spearman’s rank correlations between urinary or plasma sema6D concentrations and other parameters.

There was a statistically significant negative correlation between plasma sema6D and the age of patients (R = −0.6; *p* = 0.019), whereas no correlation with urinary sema6D. There was also a strong negative correlation between plasma sema6D and pack-years (R = −0.69, *p* < 0.0001) but no correlation with urinary sema6D (Figure 7).

We also performed a search for predictors of sema6D concentrations, but we did not obtain any significant outcomes (Table 2).

## 4. Discussion

Considering bladder cancer is a common neoplasm characterized by frequent progression and recurrence, it is of utmost importance to search for biomarkers that could aid its diagnosis or therapeutic decisions. Bladder cancer is one of the most expensive neoplasms to handle [22]. Its management is associated with periodical cystoscopies and TURBT, which are invasive and expensive, so finding a non-invasive marker that would allow the doctors to limit the cystoscopy necessity would decrease the costs of bladder cancer treatment.

Currently, there is no bladder cancer biomarker that would be routinely used in daily clinical practice. Nevertheless, there were attempts to create such tools, but still, they are not widely used in urology. An example could be the Xpert Bladder Cancer Monitor, which is a PCR-based biomarker test that is a non-invasive method using urine as a biological material [23].

Hence, our idea was to study semaphorins, not only in the blood but also in urine, which is an ideal biological material to collect due to the non-invasive and unpainful nature of the procedure. It ensures a higher probability of patients’ cooperation during the diagnostic process. Moreover, urine also seems to be a useful biological fluid that, considering it originates from the bladder, may contain different substances secreted by the tumor, which could be further analyzed. Urinary semaphorins have rarely been studied—mainly in terms of acute kidney injury [24] or diabetes nephropathy [25], so we have little data for comparison; nevertheless, it is a promising idea to apply in the daily clinical setting.

First, we examined the sema6D plasma and urinary concentration in patients and compared it to the controls. Sema6D was significantly higher in the patients only in the urine, not plasma, which may indicate its association with NMIBC occurrence. To date, only two proteins from the semaphorin family have been studied in terms of their potential involvement in bladder cancer management. The first study concerned sema3A and, similar to our experiment, was performed on urine samples, but also on bladder epithelium samples [14]. They also observed higher urinary sema3A concentrations in patients with bladder tumor. Furthermore, urinary sema3A concentration correlated with a number of present tumors. As for this finding, we did not share it, however; in our case only a small percentage of patients presented with multiple tumors. The authors also examined sema3A in tissue and found high expression in high-grade bladder cancer, moderate expression in low-grade, and hardly any expression in non-malignant tissues. However, they did not study the correlation between the urinary sema3A and tumor grading, as we did.

The second study regarding the role of semaphorins in bladder cancer was conducted by Lu et al. on sema4D. They found significantly higher expression of sema4D mRNA in cancer tissue compared to healthy bladder tissue. Moreover, sema4D was able to promote cancer cells’ viability and metastases. The mentioned semaphorin seems to activate the PI3K/AKT signal pathway in bladder cancer cells, which influences cell growth and survival, hence playing a role in tumorigenesis [15].

Considering we found higher urinary sema6D in bladder cancer patients and the observations of other scientists about the roles of semaphorins in this neoplasm, we may suspect that it promotes the development of bladder tumor; however, this requires more in-depth research.

Although several semaphorins have been found to correlate with a cancer prognosis (e.g., clear-cell renal cell carcinoma, hepatocellular carcinoma, osteosarcoma, gastric or pancreatic cancer) [13,26,27,28], we unfortunately did not share such observations. We analyzed if sema6D concentration is associated with the tumor stage, but we did not observe any correlation, either for plasma or for urinary sema6D. We also found no association between plasma or urinary sema6D and tumor grade. Hence, it cannot be used at this point for the prediction of bladder cancer staging or grading and establishing prognosis for the patients.

As for the possible association between sema6D concentration and tumor localization, we observed significantly higher sema6D concentrations in plasma of subjects with the lateral wall tumor and a strong upward trend for urinary sema6D concentration in the same localization. Considering plasma sema6D was not statistically significantly higher in patients compared to controls, this finding has limited application at this point. Urinary sema6D seems to be associated with the tumor origin in the lateral wall of the bladder; however, that should be further studied on larger cohorts. We did not manage to find any other paper that would investigate this issue.

Considering smoking is a known risk factor for bladder cancer and we indeed observed significantly more smokers among the patients than controls, we tried to look for the association between the number of pack-years and concentration of sema6D in subjects with cancer. Cigarette smoking contributes to bladder carcinogenesis due to the content of several harmful substances in the smoke, e.g., β-napthylamine, tobacco-specific nitrosamines, and 4-aminobiphenyl [29,30]. Surprisingly, plasma sema6D concentration was negatively correlated with pack-years among the patients. It could be explained in several ways. While smoking is a risk factor for bladder cancer, the degree of exposure (e.g., measured in pack-years) might influence sema6D levels in a complex manner. Moreover, there could be biological interactions between smoking-related carcinogens and sema6D expression. Smoking induces a variety of systemic effects, including inflammation and oxidative stress [31], which might alter sema6D metabolism or excretion. Clinically, perhaps sema6D could be used as a marker of higher bladder cancer incidence among patients who do not smoke much. Nevertheless, this observation has a limited application since plasma sema6D concentration was not statistically different between patients and controls. We are not aware of any other studies investigating the relationship between different semaphorins and smoking, even including lung cancer, which is most known to be associated with this risk factor.

We did not find any predictors of sema6D concentrations among the laboratory or clinical parameters.

We could not assess the potential association between the sema6D concentration and the histopathological subtype of bladder cancer considering every patient exhibited the same subtype—pure urothelial cancer. However, it is not surprising considering this is statistically the most frequent type of cancer [32].

As for the limitations of our study, it involved a relatively small number of participants with male predominance, however reflecting the epidemiology of bladder cancer. The paucity of other data on semaphorins in bladder cancer makes the discussion of our results slightly difficult. In the future, we would like to study sema6D expression in urothelial tissue.

## 5. Conclusions

In this study, we investigated for the first time the role of sema6D in bladder cancer. Plasma sema6D does not seem to be useful in the clinical practice at this point. However, the urinary sema6D concentration could potentially serve as a marker of NMIBC used for the diagnostic purposes, monitoring, and early relapse detection, or the assessment of the treatment efficacy. Urinary sema6D is probably not associated with the grading or staging of NMIBC, so it cannot be used for the prediction of disease prognosis. Tumor size and the number of tumors in the bladder do not influence sema6D urinary concentration. Sema6D may seem to be associated with the tumor origin in the lateral wall of the bladder; however, that should be further studied on larger cohorts.

## Figures and Tables

**Table 1:** Basic information about the participants.

Parameter	Controls (n = 20)	Patients (n = 40)	*p*-Value
Sex (M/F)	17/3	32/8	>0.05
Age (years)	64 ± 2.11	65.6 ± 1.46	>0.05
Creatinine (mg/dL)	0.95 ± 0.07	0.95 ± 0.04	>0.05
GFR (mL/min)	87.34 ± 4.53	85.65 ± 3.48	>0.05
Smoker (Y/N)	6/24	36/4	<0.0001
Smoking (pack-years)	8.1 ± 3.9	31.68 ± 2.45	<0.001

GFR, glomerular filtration rate; Y, yes; N, no.

**Table 2:** Potential predictors of sema6D concentration.

Parameter Estimates	Variable	Estimate	Standard Error	95% CI (Asymptotic)	|t|	*p* Value	*p* Value Summary
β0	Intercept	18.06	36.90	−99.37 to 135.5	0.4893	0.6582	ns
β1	Tumor size	−0.2445	0.6257	−2.236 to 1.747	0.3907	0.7221	ns
β2	T parameter	−0.2983	7.251	−23.37 to 22.78	0.04114	0.9698	ns
β3	Grade	−1.425	5.851	−20.04 to 17.19	0.2435	0.8233	ns
β4	Creatinine	17.95	16.65	−35.04 to 70.93	1.078	0.3600	ns
β5	GFR	0.006159	0.1360	−0.4268 to 0.4391	0.04527	0.9667	ns
β6	CRP	0.2572	0.7889	−2.253 to 2.768	0.3260	0.7658	ns
β7	Urinary sema 6D	−0.008886	0.08509	−0.2797 to 0.2619	0.1044	0.9234	ns
β8	Plasma sema 6D	−0.07721	0.05271	−0.2450 to 0.09054	1.465	0.2392	ns

ns, non-significant.

## Data Availability

Data are available from the corresponding author upon request.
